# Case of Supposed Apoplexy, without Extravasation or Rupture of Vessels

**Published:** 1822-05

**Authors:** Samuel Plumbe

**Affiliations:** Member of the Royal College of Surgeons, &c. &c.


### PATHOLOGY.

**Art. IV.**—*Case of supposed Apoplexy, without Extravasation or Rupture of Vessels.* By Samuel Plumbe, Member of the Royal College of Surgeons, &c. &c.

THE following case is submitted for insertion in the London Medical and Physical Journal, under the idea that the circumstantial evidence in favour of the opinion entertained of its character by the writer, will, when coupled with the ascertained state of the vessels of the brain, be deemed conclusive; and give it that interest in the eyes of the professional reader, which may hereafter enable him to explain some of those cases of sudden death which are often set down as inexplicable. It is a case, also, which may be reasonably suspected to be of more frequent occurrence than writers on apoplexy appear to have believed.

A female, of notorious character, aged about twenty-five, having previously enjoyed a good state of health, but exceedingly corpulent; a florid complexion, thick muscular frame, and very short neck; was found in her apartment, extended on some chairs, quite dead, within half-an-hour after having been seen in company with a man, proceeding homewards.

A report having been circulated that her death was occasioned by poison, it became necessary to examine the body. The abdomen being laid open, the stomach was exposed, and found to have no marks of mischief externally: it was tied at each extremity, and removed with its contents. The intestinal canal was examined, and presented no appearances but those of health. The aliment contained in the stomach, and the stomach itself, were also subjected to careful examination; but nothing suspicious in the former was discovered, nor did the latter exhibit any traces of inflammation: indeed it was distended with aliment, the quantity of the latter being about 1½ lbs. The woman was also in the eighth month of pregnancy.

With a state of habit and constitution of the frame decidedly indicative of an apoplectic diathesis, the original supposition as to the cause of her death having been proved to be erroneous, we were, of course, led to suspect mischief in the vessels of the brain. In addition to the external characteristics described, the state of the uterus and stomach must have contributed, at least in the recumbent position, by their pressure on the aorta, to give a more powerful impetus to the blood circulating in the brain. When, therefore, this effect was considerably augmented, (and there was no doubt, from the circumstances mentioned and the known character of the woman, that it had been so,) it seemed exceedingly probable that we should find the cause of death to have been apoplexy.

The brain was subjected to an attentive examination, and no rupture of vessel or extravasation could be discovered. The vessels were, however, in every part so gorged with blood as to warrant the idea that the effect of actual pressure on the brain had been in this way produced, and that such effect being kept up for some minutes *sub coitu*, had been the direct means of producing the extinction of life.

Since the occurrence of the above case, the writer was called to another of a somewhat similar description. This female was also about the latter end of the eighth month of pregnancy. She had been enraged with another woman, from whom she had received a blow. She fell, and was taken up perfectly comatose, and carried on her back home.

When first seen, she had the true apoplectic stertor; her pulse was beginning to get irregular, and the pupils were insensible and dilated. Her breathing was exceedingly laborious, and she was insensible to any stimulus. On being pinched, she evinced no sign of susceptibility of pain.

She was bled largely from the temporal artery and jugular vein, and her position altered so as to relieve the aorta from the pressure of the uterus; immediately after which, she began to recover. Active purgatives were given, which produced a copious effect on the bowels; and on the following morning she merely complained of pain in the head, with a little disposition to drowsiness, both of which gradually disappeared.

*65, Great Russell-street, Bloomsbury; April 6th, 1822.*

